# Patients with Severe Poststroke Fatigue Show a Psychosocial Profile Comparable to Patients with Other Chronic Disease: Implications for Diagnosis and Treatment

**DOI:** 10.5402/2011/627081

**Published:** 2011-11-09

**Authors:** Aglaia M. E. E. Zedlitz, Anne J. M. A. Visser-Meily, Vera P. Schepers, Alexander C. H. Geurts, Luciano Fasotti

**Affiliations:** ^1^Donders Centre for Cognition, Radboud University Nijmegen, 6500 HE Nijmegen, The Netherlands; ^2^Sint Maartenskliniek Research, Development and Education, 6500 GM Nijmegen, The Netherlands; ^3^Rudolf Magnus Institute of Neuroscience, University Medical Center Utrecht, 3508 GA Utrecht, The Netherlands; ^4^Center of Excellence for Rehabilitation Medicine Utrecht, Rehabilitation Center De Hoogstraat, 3583 TM Utrecht, The Netherlands; ^5^Nijmegen Centre for Evidence Based Practice, Radboud University Nijmegen Medical Centre, 6500 HB Nijmegen, The Netherlands; ^6^Department of Rehabilitation, Radboud University Nijmegen Medical Centre, Nijmegen, 6500 HB Nijmegen, The Netherlands

## Abstract

*Objective*. To obtain a psychosocial profile of patients with poststroke fatigue (PSF), which could aid in optimizing treatment strategies. *Methods*. Eighty-eight outpatients with severe PSF measured with the Checklist Individual Strength-fatigue subscale (CIS-f) and the Fatigue Severity Scale (FSS) were selected. Depression and anxiety, psychological distress, coping, social support, and self-efficacy of this group were compared to reference groups of healthy controls and patients with other chronic diseases. Associations between psychosocial characteristics and fatigue were calculated. *Results*. Compared to healthy controls, patients with PSF reported more psychological distress, less problem-focused coping, and more positive social support. Minor or no differences were found in comparison with other chronic patients. The CIS-f correlated with somatic complaints and the FSS with cognitive complaints. *Conclusion*. Patients with PSF show a psychosocial profile comparable to patients with other chronic disease. Implications for diagnosis and treatment are discussed.

## 1. Introduction

In recent years, researchers have become increasingly interested in one of the most common and persisting complaints after stroke, that is, poststroke fatigue (PSF). PSF is best described as a feeling of early exhaustion with weariness, lack of energy and aversion to effort [1] that develops during physical or mental activity and is usually not ameliorated by rest [2, 3]. The percentages of patients reporting fatigue after stroke range from 38% to 77% [4], and these percentages do not seem to decline in the chronic stage [4–6]. Furthermore, PSF appears to be related to higher mortality rates and poorer rehabilitation outcomes [7]. However, the pathogenesis of PSF is still poorly understood and, until now, effective treatments are still lacking [4, 8].

Although the mechanisms leading to PSF are still elusive, its origin is believed to be multifactorial [4]. PSF may be a direct result of organic brain damage [9], as it has often been reported in other types of brain disease, such as traumatic brain injury [10]. However, only a few associations of fatigue with biological markers have been reported in the stroke population [4]. In other words, although stroke severity may play a role, there is little evidence linking lesion size or location to PSF [3–5, 11]. The origin of PSF could also be related to psychosocial factors, which has been the focus of numerous studies (see Lerdal et al., 2009, [4] for an overview). Of all psychosocial factors, the most investigated are depression, anxiety, coping skills, social support, and self-efficacy. Nevertheless, also in this area, unequivocal findings are scarce [4].

Because fatigue is generally considered as a symptom of depression, an overlap between both phenomena seems undeniable [1, 4, 9]. Indeed, an association between fatigue and depressive symptoms is a consistent finding in the literature not only in stroke patients [2, 5–7, 11–14], but also in patients with traumatic brain injury [10] and in otherwise healthy subjects [10, 13]. Yet, in those studies that specifically looked at patients who suffer from depressive symptoms and/or fatigue, a clear dissociation between the two phenomena was also present with groups of patients reporting just fatigue or just depression [5, 6, 11, 15]. The same seems to be true for anxiety [7]. The studies that have examined coping style, self-efficacy, and social support suggest that emotion-oriented coping [2, 16], locus of control directed to powerful others [5], and lack of social support [4] are also associated with PSF. These studies are, however, few in number and cross-sectional in design and did not specifically focus on patients for rehabilitation purposes. As a result, the nature of the psychosocial profile of patients suffering from PSF is still largely unknown, providing only scant clues for optimizing the treatment of PSF [4, 8].

The aim of this study was to compare a psychological and social profile of stroke patients, with severe PSF selected for rehabilitation, to the one of healthy control subjects and patients with other chronic diseases and to investigate the strength of the association between fatigue and several psychosocial variables. Knowledge about this psychosocial profile can help to optimize PSF treatment.

## 2. Methods

### 2.1. Subjects

Data were gathered from patients with PSF who participated in a larger multicenter study (COGRAT: Effectiveness of Cognitive and Graded Activity Training on PSF [17]). In this study, 231 patients were recruited via their treating physicians and psychologists (*n* = 64), through an article about the COGRAT study in various newspapers (*n* = 151), or based on their consent for renewed contact given in earlier studies (*n* = 16). The study was approved by the local ethics committee and all eligible subjects signed an informed consent.

For the COGRAT study outpatients in the chronic stage of stroke (at least 4 months after stroke) were identified according to the following criteria: (1) age between 18 and 70; (2) last stroke episode longer than 4 months ago (either cerebral infarction, or intracerebral or subarachnoid hemorrhage; single or recurrent stroke); (3) severe fatigue (Checklist Individual Strength 20R-Fatigue subscale [18] ≥ 40); (4) ability to walk independently. Patients were excluded from the study if they had (1) severe visual hemineglect, Behavioural Inattention Test ≤ 129 [19], (2) severe memory deficits (Rivermead Behavioural Memory Test [19] screening score < 8), (3) executive impairments (Behavioural Assessment of the Dysexecutive Syndrome [19] < borderline), (4) moderate to severe aphasia (Token Task [19] >12), (5) severe cardiac or pulmonary disease, or (6) co-morbid depression (Hospital Anxiety and Depression Scale depression scale > 10). If the HADS depression scale score was between 8 and 10, a clinical interview (MINI DSM-IV [20]) was conducted to exclude patients with clinical depression.

Of the 124 excluded patients, 47 (54%) were not severely fatigued and 21 had too high levels of depression. The remaining 56 patients met one or more other exclusion criteria, such as memory deficits, mobility deficits, or aphasia. Nineteen more patients withdrew their consent before completion of assessment. Thus, 88 patients (38%) were finally included. These participants did not differ significantly with regard to age and gender (alpha = 0.05) from the nonparticipants and those excluded based on too high levels of depression.

### 2.2. Demographic and Clinical Assessment

Data on age, sex, marital status, educational level, and stroke type and side were obtained from the patients and their medical records. Severity of paresis was assessed with the Motricity Index (MI) of the affected lower extremity [21]. The MI was recorded because lower extremity paresis is strongly related to balance [22] and mobility after stroke [23], and mobility might be associated with poststroke depression [24].

### 2.3. Assessment of Fatigue

Two widely used and well-validated measures of fatigue were used in this study, the subscale fatigue of the Checklist Individual Strength 20R (CIS-f) [18] and the Fatigue Severity Scale (FSS) [25].  The CIS-f consists of 8 out of 20 items of the questionnaire, asking about fatigue severity in the two weeks before the assessment, to be indicated on a 7-point Likert scale (range 8–56). Patients with a score ≥40 on this subscale were defined as being severely fatigued [6]. In the FSS, individuals rate their agreement with nine statements on a 7-point Likert scale concerning fatigue severity, frequency, and impact on daily life. The mean score (range 1–7) is then calculated. The threshold for moderate to high impact of fatigue using the FSS is commonly set at either 4 or 5 [26].

### 2.4. Psychosocial Assessment

To study the psychosocial characteristics of patients with PSF, self-report questionnaires regarding depression, anxiety, psychological distress, coping, social support, and self-efficacy were used. Depression and anxiety were measured with the Hospital Anxiety and Depression Scale (HADS) [27]. The HADS is a 14 item self-report measure, with seven items forming a depression subscale and another seven constituting an anxiety subscale. Each item is rated on a four-point scale, ranging from 0 to 3, with 3 reflecting the highest distress. Total scores for each subscale range from 0 to 21 and are categorized as normal (0–7), mild (8–10), moderate (11–14), or severe (15–21) [28].

The Symptom Checklist-90 (SCL) [29] measures psychological distress [30]. The scale consists of 90 items scored on a 5-point Likert scale. Nine psychopathology scores can be derived and the total score (GSI) reflects a global severity index of psychological distress. Furthermore, a personality severity index (PSI) to assess personality problems can be calculated [31]. This is done by transferring raw scores to SCL-90R scales, and then comparing the mean scores of the scales interpersonal sensitivity, hostility, and paranoid ideation to the mean value of the remaining scales.

Coping strategies were assessed with the coping inventory for stressful situations (CISS) [32]. Forty-eight items are scored on a 5-point Likert scale, resulting in three subscales: Problem-focused coping, Emotion-focused coping, and Avoidance-focused coping.

Social support was assessed with the Social support list (SSL-12) [33] that consists of twelve statements regarding perceived positive and negative social support from the primary social network. Positive support is described by three subscales: everyday support, support in problem situations, and esteem support.

The Self-Efficacy Scale (SES) was used to assess the sense of control in relation to fatigue complaints [34]. It consists of 5 statements each scored on a 5-point scale. The total scores ranges from 5 to 25, with a higher score reflecting more sense of control.

### 2.5. Data Analysis

Descriptive statistics (i.e., mean and standard deviation (SD)) were calculated for all psychosocial characteristics. These values were compared to known reference values and derived Z-scores from healthy controls and patient populations (General practice, traumatic brain injury, chronic pain, Multiple Sclerosis, Parkinson, Cancer, Rheumathoid Arthritis, and Chronic Fatigue Syndrome) reported in the literature [28, 29, 32, 33, 35–37]. Based on these Z-scores different categories were labeled as follows: < −1.28 = very low, <−.84 = low, <−.525 = below average, between −.525 and  .525 = average, >.525 = above average, >.84 high and >1.28 very high.

The presence of personality problems was determined by calculating a PSI from the raw scores of the SCL. Associations between the CIS-f-FSS fatigue scales and gender, marital status, lesion characteristics, and PSI were calculated with *χ*_2_ analyses. Spearmen rank correlations were calculated with the ordinal demographical, stroke, and psychosocial characteristics. Then a stepwise multiple regression analysis was performed on the variables significantly associated with fatigue to establish their unique contribution. All data analyses were computed with SPSS version 17.0, using Holm's correction to adjust for multiple analyses [38].

## 3. Results

Demographic data, severity of paresis, fatigue scores, and stroke characteristics are summarized in Table 1. Scores on both fatigue scales indicated on average “severe fatigue.” On the CIS-f, 92.0% of the subjects scored above 40. On the FSS, 92.0% scored above 4 and 69.3% above 5. The mean MI was 90.2 indicating on average mild lower extremity paresis. Mean postonset time since last stroke was 4.3 years, which did not differ significantly between single and recurrent strokes.

### 3.1. Psychosocial Characteristics

To investigate the psychosocial characteristics of our patients, the scores were compared to reference values obtained from norm groups of healthy controls and patient groups from general health practice and with various chronic afflictions (Table 2). The HADS anxiety scores were comparable to other patient groups but high when compared to the general population. Using the Z-score derived, abovementioned categories [28], 60.2% of the patients had normal, 17% mild, 18.2% moderate, and 4.5% severe scores on HADS-anxiety. On the depression subscale of the HADS, we found high scores compared to both the general population and general practice patients. Still, 64.8% of our subjects had normal and the remaining 35.2% only mild depressive symptoms. The scores on the SCL were generally high compared to healthy controls, but in comparison to other patient groups the SCL scores were average, except for the subscale “Obsessive Compulsive” which was above average, reflecting more subjective cognitive complaints. The PSI is a categorical value and could therefore not be compared to reference values. Nevertheless, the incidence rate indicated that 93.2% of the patients were free from personality problems.

As for coping strategies, our patients showed slightly lower levels of problem-focused and avoidance strategies than healthy controls. However, in comparison to other patient groups, they scored average or above average for all strategies. Furthermore, our patients received more positive social support with as many negative social interactions compared to healthy controls and other patient groups. Compared to patients with chronic fatigue syndrome (CFS) [37], they reported to be “more in control” when assessed with the SES.

### 3.2. Associations of Demographic, Clinical, and Psychosocial Characteristics with Fatigue

Table 3 shows the correlation coefficients of demographic, clinical, and psychosocial characteristics with both fatigue scores. No associations with either fatigue scale (CIS-f or FSS) were found for demographic data, stroke characteristics, or severity of paresis (MI).

The CIS-f scale showed a statistically significant but moderate association with SCL-somatic and SCL-depression. The FSS was only related to the Obsessive Compulsive subscale of the SCL. No other significant associations were found for any psychosocial measure.

Multiple regression analysis was performed only for the CIS-f with associated variables, since the FSS correlated with just one psychosocial variable (Table 3). SCL-Som significantly predicted CIS-f scores, *β* = .54, *t*(86) = 5.89, *P* < .001. SCL-Dep did not add significantly to the model (*P* = .78) with no concerns for multicolinearity (VIF = 1.56, tolerance =  .64).

## 4. Discussion

The aim of this investigation was to obtain a psychosocial profile of patients suffering from severe PSF in order to tease out options for the rehabilitation of fatigue. The results of this study suggest, however, that these patients are not characterized by a distinct psychosocial profile. In comparison to healthy controls, PSF-patients reported high psychosocial distress, high positive social support, and low problem-focused coping. However, compared to other chronic patient groups, we found no marked discrepancies with regard to distress, coping styles, social support, and self-efficacy. Moreover, independent associations with fatigue were only found for SCL-Somatic Complaints with CIS-f (*r* = .54; *P* < .001) and SCL-Obsessive Compulsive with FSS (*r* = .35; *P* < .001), but not for any other psychosocial variable or stroke characteristic (e.g., time since stroke, single versus recurrent, nature of lesion, and lesion side).

The subscale Obsessive Compulsive of the SCL might mimic the direct neurological consequences of stroke, since it includes items such as “trouble with thinking,” “mental slowness,” “needing to check things” and “thinking things over.” Although no direct association between fatigue severity and severity of cognitive deficits has been found [4, 5, 14], a relationship between fatigue and cognitive complaints, such as mental slowness, has been substantiated [13, 39].

A probable cause for the experienced fatigue may be found in this light. Cognitive deficits may temporarily be compensated for by exerting increased mental effort, which then may cause fatigue [40]. Indeed, widespread brain activity has been shown in patients with unilateral lesions of one cerebral hemisphere trying to perform a unimanual task with their affected hand [41]. Such activities typically require a great amount of attentional resources, because subjects act at the limits of (or even beyond) their functional capacities [42]. A parallel can be drawn from studies of traumatic brain injury where affected individuals showed more dispersion and more brain regions activated when engaged in an attention task than healthy controls [43, 44].

Thus, PSF might be associated with underlying cognitive mechanisms that are independent of the extent of the brain lesion and of psychopathological problems related or unrelated to stroke. If this notion is valid, it would be consistent with the finding that fatigue is almost as frequent in relatively mildly affected patients as in the more severely affected ones [4, 5], because it will become a problem whenever subjects try to overcome their individual subtle or severe limits. As soon as subjects are able to deal with their functional limitations more effectively, taking into account their limited attentional capacity and mental energy, fatigue may gradually become less severe. 

The association between fatigue and somatic complaints could also be seen in line with this hypothesis. Somatic complaints may reflect the direct physical consequences of stroke, causing functional limitations and/or pain, requiring more task-related physical and mental effort and thereby provoking fatigue. Another part of the somatic complaints could be explained as the physical expression of fatigue. For example, many patients report a heavy feeling in arms and legs, nausea, or headaches when becoming tired.

As in previous studies, we first found an association between depression and fatigue on one of our fatigue scales [2, 5–7, 11–14]. However, this association was subordinated to somatic complaints in the regression analysis. A variant of this moderating effect has been previously reported in a study wherein the association between fatigue and depression became weaker, when controlled for by sickness and impact on ambulation [6]. This finding emphasizes not only the dissociation of fatigue and depression, but also the need to assess physical complaints in depth.

A rather unexpected but noteworthy finding of this study was that both fatigue measurements used, the FSS and CIS-f, were associated with completely different variables as shown in Table 3. The use of more than one fatigue scale in clinical practice and research might therefore be warranted. 

This study holds several limitations. Due to its cross-sectional design we are unable to infer causal relationships. Furthermore, our inclusion criteria restricted the variability of fatigue and mobility, thereby possibly lowering associations. The exclusion of patients with severe cognitive deficits, depression and motor problems might be considered both a strength and a limitation of this study. The major limitation is that it precludes generalization to other stroke patients. On the other hand, this choice enabled us to single out the relationship between fatigue and psychosocial factors, without the influence of these confounding factors.

## 5. Conclusions and Implications for Treatment

By comparing our study group to other reference groups, PSF patients displayed a “normal chronic patient” psychosocial profile. Only cognitive and somatic complaints were associated directly with fatigue. We therefore propose that (a part of) the fatigue might be a consequence of the inadequate adaptation to diminished and or less efficient attentional resources after stroke.

Our findings suggest the following implications for treatment. Somatic complaints should be directly addressed, whenever possible. Graded physical activity programs might be an important contribution to the treatment of PSF, since exercise has been found to be helpful in improving physical and functional outcomes and to reduce fatigue in various other medical conditions [45, 46]. Such programs might help stroke patients to gradually increase physical activity without experiencing distressing bodily symptoms.

Second, cognitive compensation strategies circumventing the limited energetic resources available to patients suffering from PSF might also be beneficial. These compensation strategies could entail an enhanced planning and variation of activities to foster a more regular pattern of activity and rest [14]. Here, patient education and goal setting could be added to improve patient motivation and adherence [47].

Third, since symptoms of depression and anxiety are common in PSF patients, it is important to address these when present, since they may compromise self-management [48]. An augmented form of cognitive behavioral therapy, as proposed by Broomfield et al., 2010, [49], is a good starting point to address these issues. It takes into account cognitive deficits and grievance of loss of abilities and could also aid in implementing the behavioral changes needed to apply compensation strategies. Lastly, our results point to the use of more than one scale to assess PSF, highlighting that fatigue is not only a common but also a complex and multifaceted syndrome.

## Figures and Tables

**Table 1:** Demographic data, severity of paresis, stroke data and fatigue scores (*n* = 88).

*Demographics *	
Age, mean (SD)	54.6 (8.8)
Gender, male, *no.* (%)	46 (52.3%)
Living together/married, *no. *(%)	71 (80.7%)
Educational level, median (SD), (1 = low to 7 = high)	5 (1.2)
*Severity of paresis*	
Motricity Index, mean (SD)	90.2 (13.6)
*Stroke*	
Time since last lesion, mean (SD)	4.3 (5.3)
Single Stroke, *no*. (%)	67 (76.1%)
Ischemic stroke^a^, *no.* (%)	64 (72.7%)
Hemorrhage^b^, *no. *(%)	6 (6.82%)
Subarachnoid hemorrhage, *no. *(%)	9 (10.2%)
Mixed^c^, *no. *(%)	4 (4.5%)
*Fatigue*	
CIS-f, mean (SD)	45.4 (5.6)
FSS, mean (SD)	5.2 (1.0)

CIS-f: Checklist Individual Strength subscale fatigue.

FSS: Fatigue Severity Scale.

^a^ICVA category: 18 left hemispheric, 44 right hemispheric, 4 infratentorial, and 3 bilateral.

^b^Hemorrhage category: 4 right hemispheric, 2 bilaterial.

^c^Mixed: 4 infratentorial and other lesion.

**Table 2:** Means and standard deviations of baseline psychosocial characteristics of PSF patients in comparison with healthy controls and patients with other chronic disease (*n* = 88).

Scale at inclusion	Mean (SD) Study group	In comparison to:		
		*Healthy controls*	*Patient groups*	
*Psychosocial characteristics*				

HADS		*General population 57–65 years *(*n* = 1901) [35]	*General practice patients *(*n* = 112) [35]	*Traumatic brain injury patients *(*n* = 100) [28]
Anxiety	7.27 (3.76)	High	Average	Average
Depression	7.05 (2.37)	High	High	Average

Psychological distress: SCL		*Healthy controls *(*n* = 2092) [29]	*General practice patients *(*n* = 920) [29]	*Chronic pain patients *(*n* = 2461) [29]
Anxiety	15.08 (4.84)	Above average/high	Average	Average
Phobic anxiety	9.13 (3.06)	Above average/high	Average	Average
Depression	27.44 (7.80)	High	Average	Average
Somatic	22.25 (6.61)	High	Average	Average
Obsessive-compulsive	20.51 (6.47)	Very high	Above average	Above average
Interpersonal sensitivity	26.98 (8.95)	Average	Average	Average
Hostility	8.36 (2.45)	Average	Average	Average
Sleep disturbances	6.74 (3.21)	Above average/high	Average	Average
Total (GSI)	149.00 (35.80)	High	Average	Average

Coping styles (CISS)		*Working adults *(*n* = 683) [32]	*Multiple Sclerosis patients *(*n* = 96) [32]	*Parkinson patients *(*n* = 75) [32]
Problem focused	51.89 (10.98)	Low	Average	Above average
Emotion-oriented coping	35.75 (10.51)	Average	Average	Above average
Avoidance	40.55 (10.66)	Below average	Above average	Above average

Social support (SSL-12)		*Healthy elderly *(*n* = 5279) [33]	*Cancer patients *(*n* = 475) [36]	*Rheumatoid arthritis patients *(*n* = 246) [36]
Everyday support	10.69 (2.08)	Above average		
Support in problem situations	9.76 (2.24)	High		
Esteem support	10.35 (2.10)	High		
Negative social interactions	9.91 (2.92)	Average [36]	Average	Average

Self-efficacy (SES)			*Chronic fatigue syndrome patients *(*n* = 292) [37]	
Self efficacy	16.57 (3.32)		Above average	

HADS: Hospital Anxiety and Depression Scale, SCL: Symptom Checklist-90, CISS: Coping Inventory for Stressful Situations, SSL-12: Social Support List, and SES: Self Efficacy Scale.

Reference values were derived from known norm groups or research data from different studies. From research data, means and Z-scores were calculated, and categories were described based on Z-scores as follows: <−1.28 = very low, <−.84 = low, <−.525 = below average, between −.525 and  .525 = average, >.525 = above average, >.84 high, and >1.28 very high.

**Table 3:** Correlation coefficients of demographic, clinical, and psychosocial characteristics with PSF (*n* = 88).

	CIS-f	FSS
* Demographic data*		
Age^2^	n.s.	n.s.
Gender^1^	n.s.	n.s.
Marital status^1^	n.s.	n.s.
Educational level^2^	n.s.	n.s.

*Stroke characteristics*		
Single or recurrent stroke^1^	n.s.	n.s.
Lesion side of last stroke^1^	n.s.	n.s.
Time since last stroke^2^	n.s.	n.s.

*Severity of paresis (MI)* ^2^	n.s.	n.s.

Psychosocial characteristics		

*Anxiety and depression (HADS)* ^2^		
HADS-anxiety	n.s.	n.s.
HADS-depression	n.s.	n.s.

*Psychological distress (SCL)* ^2^		
Anxiety	n.s.	n.s.
Phobic anxiety	n.s.	n.s.
Depression	0.35*	n.s.
Somatic	0.53*	n.s.
Obsessive compulsive	n.s.	0.36*
Intrapersonal sensitivity	n.s.	n.s.
Hostility	n.s.	n.s.
Sleep disturbances	n.s.	n.s.
Total (GSI)	0.34*	n.s.

Personality problems (PSI)^1^	n.s.	n.s.

*Coping (CISS)* ^ 2^		
Problem-focused coping	n.s.	n.s.
Emotion-oriented coping	n.s.	n.s.
Avoidance	n.s.	n.s.
Distraction seeking	n.s.	n.s.
Company seeking	n.s.	n.s.

*Social support (SSL-I-N)* ^2^		
Total positive support	n.s.	n.s.
Negative social interactions	n.s.	n.s.

*Self-efficacy (SES)* ^2^	n.s.	n.s.

^1^ *χ* ^2^ analyses (categorical data)

^2^Spearman rank correlation coefficients

n.s.: not significant

*significant at Holm's correction (*P* < .0011)

CIS-f: Checklist Individual Strength fatigue severity subscale

FSS: Fatigue Severity Scale
